# Evaluation of inducible promoter–riboswitch constructs for heterologous protein expression in the cyanobacterial species Anabaena sp. PCC 7120

**DOI:** 10.1093/synbio/ysab019

**Published:** 2021-09-01

**Authors:** Jessee Svoboda, Brenda Cisneros, Benjamin Philmus

**Affiliations:** College of Chemical, Biological, and Environmental Engineering, Oregon State University, Corvallis, OR, USA; Department of Pharmaceutical Sciences, Oregon State University, Corvallis, OR, USA; College of Chemical, Biological, and Environmental Engineering, Oregon State University, Corvallis, OR, USA; Department of Pharmaceutical Sciences, Oregon State University, Corvallis, OR, USA

**Keywords:** cyanobacteria, riboswitch, inducible protein production, theophylline

## Abstract

Cyanobacteria are promising chassis for synthetic biology applications due to the fact that they are photosynthetic organisms capable of growing in simple, inexpensive media. Given their slower growth rate than other model organisms such as *Escherichia coli* and *Saccharomyces cerevisiae*, there are fewer synthetic biology tools and promoters available for use in model cyanobacteria. Here, we compared a small library of promoter–riboswitch constructs for synthetic biology applications in *Anabaena* sp. PCC 7120, a model filamentous cyanobacterium. These constructs were designed from six cyanobacterial promoters of various strengths, each paired with one of two theophylline-responsive riboswitches. The promoter–riboswitch pairs were cloned upstream of a chloramphenicol acetyltransferase (*cat*) gene, and CAT activity was quantified using an *in vitro* assay. Addition of theophylline to cultures increased the CAT activity in almost all cases, allowing inducible protein production with natively constitutive promoters. We found that riboswitch F tended to have a lower induced and uninduced production compared to riboswitch E for the weak and medium promoters, although the difference was larger for the uninduced production, in accord with previous research. The strong promoters yielded a higher baseline CAT activity than medium strength and weak promoters. In addition, we observed no appreciable difference between CAT activity measured from strong promoters cultured in uninduced and induced conditions. The results of this study add to the genetic toolbox for cyanobacteria and allow future natural product and synthetic biology researchers to choose a construct that fits their needs.

## 1. Introduction

Cyanobacteria are photosynthetic, Gram-negative bacteria that are found in both terrestrial and aquatic environments. They are known producers of a variety of bioactive and structurally unique secondary metabolites (natural products), many of which have the potential for development into new pharmaceuticals or to serve as lead compounds in drug discovery (1). Bioactive secondary metabolites have been discovered from cyanobacterial strains isolated from all environments. The recent explosion in genomic sequencing has shown that cyanobacterial genomes contain orphan biosynthetic gene clusters (BGCs), which are defined as gene clusters that are not associated with the production of a particular compound (or family of compounds). This raises the possibility that undiscovered secondary metabolites are present in secondary metabolomes produced by cyanobacteria (2).

Many strains of cyanobacteria, including the native producers of many of these secondary metabolites, grow very slowly and lack tools for genetic manipulation. The difficulty of growing many cyanobacterial strains in laboratories has led to synthetic biology tool developments in cyanobacteria lagging behind those of other more easily manipulated bacterial strains (e.g. *Escherichia coli, Streptomyces* sp. and *Saccharomyces cerevisiae*). Historically, the most common cyanobacterial host used in synthetic biology research has been *Synechocystis* sp. PCC 6803, while *Synechococcus elongatus* PCC 7942 (3), *Synechococcus* sp. PCC 7002, *Synechococcus elongatus* UTEX 2973 and *Anabaena* sp. PCC 7120 have also been used in synthetic biology applications (4). With recent advances in genome editing technologies in cyanobacteria, synthetic biology research is primed to increase (5).

*Anabaena* sp. PCC 7120 (hereafter referred to as *Anabaena* 7120) has been used to investigate cellular differentiation and nitrogen fixation. Recently, our laboratory has demonstrated that *Anabaena* 7120 is a viable heterologous host strain for the production of cyanobacterial secondary metabolites as it is able to recognize promoters from different marine cyanobacterial species (6). More recently, we have utilized combinatorial biosynthesis in *Anabaena* 7120 to produce known and unknown compounds (7), and the Gerwick and Golden laboraotries have successfully expressed cryptomaldamide in *Anabaena* 7120 (8). *Anabaena* 7120 also grows relatively quickly in synthetic media compared to other filamentous cyanobacteria, is easy to cultivate and has established protocols for genetic manipulation, making it a suitable choice as a general heterologous expression host for cyanobacterial secondary metabolites (6).

In *Anabaena* 7120, there are four main promoters utilized in synthetic biology applications. The *conII* promoter (from *E. coli*) has been used and is recognized as a strong promoter of gene expression (9). The other three promoters come from *Anabaena* 7120 and are the *nir*, *glnA* and *petE* promoters. The *glnA* promoter (P*_glnA_*) is a strong constitutive promoter (10), P*_nir_* is a strong promoter induced in nitrate-containing media but is repressed in ammonia-containing media (11) and P*_petE_* is a copper-inducible promoter (12). Despite the usefulness of a Cu-inducible promoter for tuning gene expression, P*_petE_* can be difficult to use as an inducible promoter because producing copper-free BG-11 media containing sodium nitrate as the nitrogen source (BG-11(Nit) media) is arduous (requires Milli-Q water >18 Ω, use of plastic vessels and avoiding autoclaving for sterilization). To further develop *Anabaena* 7120 as a heterologous host used in the production of secondary metabolites, biofuels, commodity chemicals or heterologous proteins, new ‘synthetic’ promoters are needed. In this report, we characterize six different promoters in combination with two theophylline-sensing riboswitches as new tools for synthetic biology in *Anabaena* 7120.

Riboswitches are sequences of nucleic acids that regulate protein expression by forming secondary structures that are altered in the presence of a specific ligand (13). The first riboswitches were identified in bacterial vitamin pathways and sensed thiamin (14), thiamin diphosphate (14, 15), riboflavin (15) and coenzyme B_12_ (16). They work at either a transcriptional or translational level, by either inducing or repressing expression (13). Topp *et al.* developed five translational-level, expression-inducing synthetic riboswitches for bacteria that respond to theophylline, a caffeine analog (17). While naturally occurring riboswitches are a native genetic regulation system in many bacterial species, various known natural riboswitches cannot be used as synthetic biology tools in cyanobacteria because their ligands are key metabolic intermediates (18). Synthetic riboswitches have the benefit of not interfering with native regulatory systems because the synthetic ligand is not recognized by the organism (17). Translational-level riboswitches work via a messenger RNA (mRNA) sequence that binds to itself, sequestering the ribosome-binding site either in the presence or in the absence of a ligand, which prevents translation until the conformation changes by either binding or unbinding the ligand. Riboswitches therefore allow inducible control with natively constitutive promoters (18).

Nakahira *et al.* demonstrated that the riboswitches from Topp *et al.* worked in the cyanobacterium *Synechococcus elongatus* PCC 7942 and developed a variant that was optimized for cyanobacteria (19). These riboswitches have since been successfully used in various cyanobacterial species: to regulate glycogen metabolism in *Synechococcus elongatus* (20); to build NOT-gate molecular circuits in *Anabaena* 7120, *Synechocystis* sp. PCC 6803, *S. elongatus, Leptolyngbya* BL0902 and *Synechocystis* WHSyn (21); and to regulate expression of heterologous proteins in *Synechocystis* sp. PCC 6803 (22). Ma *et al.* tested all six theophylline-responsive riboswitches in diverse cyanobacterial species, including *Anabaena* 7120 (23). Of the riboswitches tested, riboswitch E and riboswitch F were shown to have the highest induction ratios in *Anabaena* 7120. Riboswitch E had a higher induced production of the reporter yellow fluorescent protein (YFP) but also a higher baseline, whereas riboswitch F effectively prevented translation in the absence of theophylline but also had a relatively low induced production compared to riboswitch E–containing constructs (23). More recently, Higo and Ehira engineered a strain of *Anabaena* 7120 to produce 1-butanol under photosynthetic conditions. Two transcriptional level riboswitches (from Ceres *et al.* (24).) were combined with heterocyst-specific promoters to allow for production to be induced exclusively in the non-oxygenic microenvironment of the nitrogen-fixing heterocyst cells (25, 26).

In this report we design, synthesize and test promoter–riboswitch constructs in *Anabaena* 7120. We chose five promoters from marine cyanobacteria of varying strengths and the *petE* promoter from *Anabaena* 7120 paired with either riboswitch E or riboswitch F from Ma *et al.* (23). These 12 promoter–riboswitch constructs were cloned upstream of a chloramphenicol acetyltransferase (*cat*) gene to provide a quantitative reporter assay to measure protein production.

## 2. Materials and methods

### 2.1 General methods

*Escherichia coli* strains were routinely grown in Lysogeny broth, Miller (LB) at 37°C at 200 rpm. Kanamycin was supplemented at 50 μg/ml, chloramphenicol was supplemented at 30 μg/ml and spectinomycin was supplemented at 100 μg/ml for growth and selection of *E. coli*. Spectinomycin and streptomycin were supplemented at 2.5 μg/ml each for selection and maintenance of *Anabaena* 7120. Acetyl CoA, lithium salt; 5,5′-dithiobis(2-nitrobenzoic acid) (DTNB); and cat protein were purchased from MilliporeSigma. Theophylline was dissolved at an initial concentration of 200 mM in dimethylsufloxide (DMSO) by heating in a 42°C water bath until all the solid material is dissolved. All other chemicals were obtained from VWR and used without further purification. All microbial culturing and transfers were performed using the aseptic microbial technique.

#### Plasmid design and synthesis.

Promoter and riboswitch sequences were designed by the Philmus lab using Gene Construction Kit and synthesized by the Joint Genome Institute (JGI). The synthesized DNA sequences were ligated into pPJAV561, which had been digested with SmaI. The ligation mixture was transformed into *E. coli*, and positive colonies were selected on LB agar containing spectinomycin. Plasmids were isolated and sequenced to verify insert sequence and directionality. Correct plasmids were shipped to the Philmus lab as frozen glycerol stocks of *E. coli*.

#### Biological culturing.

*Escherichia coli* was grown in LB media supplemented with spectinomycin at 100 μg/ml for plasmid selection. *Anabaena* 7120 was routinely grown in BG-11(Nit) medium supplemented with spectinomycin and streptomycin at 2.5 μg/ml each. *Anabaena* 7120 cells were cultivated in a Hoffman incubation chamber (Hoffman Manufacturing, Inc, Corvallis, OR, USA) at 28°C under 24 h constant light illumination, with the light being supplied by two GE Lighting 49893 F40/PL/AQ Plant and Aquarium Tube bulbs with a light intensity of 25 microeinsteins/m^2^/s, and the atmosphere contained 1% CO_2_. Liquid cultures were shaken at 220 rpm. Plasmids were conjugated into *Anabaena* 7120 from *E. coli* as previously described (6).

Starter cultures of *Anabaena* 7120 were inoculated by scraping cells from a solid media plate into 3 ml liquid media and grown at 28°C and 220 rpm in a 1% CO_2_ atmosphere. After 5–7 days of growth, 250 μl of starter culture was added to 3 ml of fresh media for the production cultures. Production cultures grew for 1 week before induction or washing and then were given 1 day under experimental conditions before harvesting.

Cultures were induced by adding 30 μl of 200 mM theophylline in DMSO for a final concentration of 2 mM theophylline or 30 μl DMSO for the control condition, before returning to the incubator to shake for 24 h before harvesting. Removal of theophylline was accomplished by transferring cultures to centrifuge tubes and spinning in an Eppendorf 5810R centrifuge for 20–30 min at 1200 × *g*, aspirating out the supernatant and rinsing the original culture tubes with 1 ml of fresh media to pour into the respective centrifuge tubes to resuspend the cell pellets. This was repeated for three total rinse cycles, after which the cells were transferred back into the rinsed culture tubes containing 3 ml of fresh media, before returning to the shaker to grow for 24 h before harvesting. The fresh media was either BG-11(Nit) with 2 mM theophylline (in 30 μl DMSO) or BG-11(Nit) with 30 μl DMSO as per the experimental conditions. The 0-h timepoint for the wash experiment was taken by removing 1 ml of culture from the culture tubes immediately after washing and then pelleting those cells via centrifugation for 20–30 min at 1200 × *g*. The time course was conducted by removing an additional 1 ml of culture from each of the culture tubes at 24 h post-induction and pelleting the cells via centrifugation for 20–30 min at 1200 × *g* in an Eppendorf 5424R microcentrifuge.

Cells were harvested by transferring 1.5 ml of the cultures into microcentrifuge tubes and pelleting the cells using centrifugation at 1200 × *g* for 20–30 min in an Eppendorf 5424R microcentrifuge. The media was removed by aspiration, and the procedure was repeated until all the cells were collected. The cell pellets were stored at −80°C until the CAT assay was performed.

#### Chloramphenicol acetyltransferase assay.

CAT assays followed the methods described previously with the following modifications (6). Harvested cell pellets were resuspended in 250–500 μl of 10 mM Tris base (pH 7.8). Cell suspensions were sonicated 2–4 times for 10 s with a Qsonica CL-188 sonicator at 30% amplitude and then centrifuged for 10 min at 4°C and 21 000 × *g*. The absorbance at 620 nm of the supernatant was measured with a BioTek Synergy 4 plate reader and used to dilute to a concentration of A_620_ = 0.05 with 10 mM Tris, after the first induction experiment showed that undiluted supernatant contained elevated CAT activity that prevented the acquisition of sufficient points to calculate an accurate slope. Lysates for cells containing pJGI005, pJGI006, pJGI011 and pJGI012 were diluted to A_620_ = 0.005. A portion (5 μl) of these dilutions were placed in the appropriate wells of a 96-well polystyrene clear bottom plate (Costar 3370, Corning, Tewksbury, MA, USA). The samples were combined with 95 μl of the CAT assay reaction mixture (87.8 μl Tris (100 mM, pH 7.8), 2.9 μl acetyl-CoA (5 mM in water), 2.9 μl DTNB (2.5 mM in water), 1.4 μl chloramphenicol (0.3% w/v in 95% aq. ethanol)) using a Rainin Liquidator 96 LIQ-96-200. The plate was then transferred to a BioTek Synergy 4 plate reader, and absorbance at 412 nm was measured every 30 s for 30 min. Linear regressions were fit to the timed data from each well, and units of CAT were quantified based on a standard curve measured. This data analysis was performed using R using a custom written code (Supplemental file 2).

## 3. Results and discussion

We previously demonstrated that *Anabaena* 7120 can recognize promoters of various strengths from different BGCs from marine cyanobacteria (Table 1) (6). This was measured by placing the *cat* reporter gene under the control of these promoters on plasmids that were then conjugated into *Anabaena* 7120. The produced CAT was quantified by a colorimetric assay (27) and confirmed through reverse transcription polymerase chain reaction that mRNA levels correlated to CAT activity (6). We chose these constitutive promoters as the sequences are not derived from *Anabaena* 7120, unlike P*_nir_* and P*_glnA_*, and can therefore be utilized in genomic integration experiments with reduced off-target integration. P*_petE_* was included because the Cu-responsive nature of P*_petE_* is sensitive enough to be induced by the low copper concentration in our usual media. These promoters alone are not responsive to theophylline (Supplementary Figure S1). We noted that the empty vector (pPJAV361) and the vector that contains a promotorless *cat* gene (pPJAV561) have negligible measurable CAT activity (Supplementary Figure S2).

**Table 1.** Cyanobacterial promoters tested by Videau *et al.* (6) and shown to be recognized by *Anabaena* 7120

Promoter	Native species	BGC	Strength in Anabaena
P*_patA_*	*Prochloron didemni*	Patellamide BGC	Weak
P*_ltxA_*	*Moorea producens*	Lyngbyatoxin BCG	Weak
P*_ltxD_*	*M. producens*	Lyngbyatoxin BCG	Medium
P*_petE_*	*Anabaena* 7120	Plastocyanin	Medium-strong
P*_curA_*	*M. producens*	Curacin A BGC	Strong
P*_barA_*	*M. producens*	Barbamide A BGC	Strong

Weak promoters displayed <1 CAT units/mg protein; medium promoters displayed 1–5 CAT units/mg protein; medium-strong promoters displayed 5–10 CAT units/mg protein and strong promoters displayed >10 CAT units/mg protein in liquid BG-11(Nit) media.

**Figure 1.**
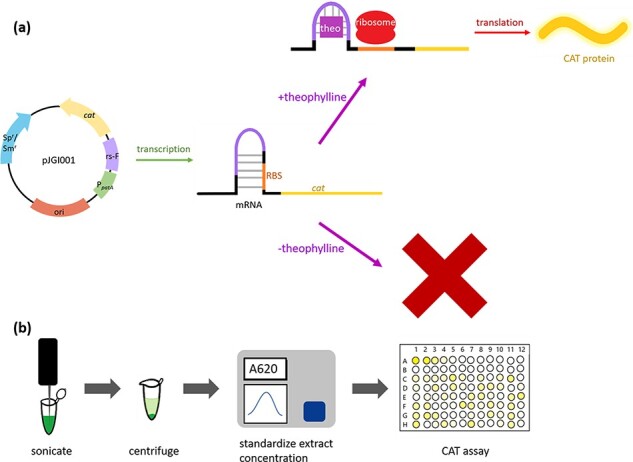
(a) Translational regulation of the production of CAT activity in *Anabaena* 7120 cells when expressed from a plasmid containing a promoter–riboswitch construct. pJGI001 drawn as an example; promoter or riboswitch varies in the different constructs (see Table 2). (b) Sample preparation workflow for the CAT assay after *Anabaena* 7120 cells have been grown and harvested as described in Section 3.1.

Our promoter–riboswitch constructs were designed so that each of these promoters were paired with each of the two riboswitches (E and F) previously found to have the highest induction ratios in *Anabaena* 7120 and other cyanobacteria (23). Ma *et al.* found that riboswitch E showed the highest induced fluorescence but also a high basal fluorescence level when not induced, while riboswitch F showed a lower level of induced fluorescence but low basal fluorescence when paired with P*_conII_* from *E. coli* using YFP as the reporter (23). The 12 promoter–riboswitch pairs (6 promoters × 2 riboswitches) were commercially synthesized and cloned into pPJAV561 (6) by the JGI to generate the 12 plasmids detailed in Table 2, using CAT as a reporter (Figure 1a). These constructs were then introduced into *Anabaena* 7120 via conjugation; the resulting mutants were grown as described in *Biological culturing*. The cells were pelleted, cell pellets were lysed via sonication and the CAT activity was measured via a colorimetric assay (Figure 1b).

**Table 2.** Plasmids used in this study

Plasmid	Promoter	Riboswitch
pJGI001	P*_patA_*	F
pJGI002	P*_petE_*	F
pJGI003	P*_ltxD_*	F
pJGI004	P*_ltxA_*	F
pJGI005	P*_curA_*	F
pJGI006	P*_barA_*	F
pJGI007	P*_patA_*	E
pJGI008	P*_petE_*	E
pJGI009	P*_ltxD_*	E
pJGI010	P*_ltxA_*	E
pJGI011	P*_curA_*	E
pJGI012	P*_barA_*	E

### 3.1 Optimizing experimental conditions

We first looked at the effect of culture volume on measured CAT activity. For this experiment, 3 and 30 ml cultures of the *Anabaena* 7120 strain containing pJGI002 were grown for 1 week in BG-11(Nit) media. Either theophylline in DMSO was added to the cultures for a final concentration of 2 mM theophylline, or an equal volume of DMSO was added to the cultures. Twenty-four hours after induction, cells were harvested from 3 ml of each culture and frozen at −80°C until CAT activity was assayed. When normalized to chlorophyll absorption (A_600_), both culture sizes (3 vs 30 ml) had a similar variation, but the 3 ml cultures showed a higher level of CAT activity (7.86 units of CAT activity per A_600_ vs 6.23 units of CAT activity per A_600_, Supplemental Figure S3a). Given these results, we decided to move forward with 3 ml cultures, which allowed all strains in a specific experiment to be grown simultaneously, given space constraints in the incubator.

In our original reports, we standardized CAT activity to milligrams of protein as determined by A_280_; however, the use of theophylline as an inducer prevented this, as theophylline has a large absorption at 280 nm. Further, because *Anabaena* 7120 is filamentous, it can be difficult to consistently measure OD_750_ for cell growth. We initially attempted to standardize CAT activity per culture volume, but this led to inconsistent results because of variation in culture growth. A direct comparison on the same samples of pJGI002 found much lower variation when standardizing to chlorophyll absorption than culture volume (Supplemental Figure S3). Plasmid pJGI002 was chosen as our test case as it is a low-to-moderate-strength construct resulting in easily measured CAT activity and showed a statistically significant increase in CAT activity when induced with theophylline. In all experiments reported below, we diluted our cell lysate to a normalized absorbance at 620 nm, which corresponds to chlorophyll absorption, as this had the greatest sensitivity. We utilized 5 μl of lysed cell supernatant diluted to an A_620_ of 0.05 for pJGI001-004 and pJGI007-010. Due to the large levels of CAT activity displayed by pJGI005, pJGI006, pJGI011 and pJGI012 at A_620_ of 0.05, these samples were diluted to A_620_ of 0.005 to ensure that the measured CAT activity fell within the range of CAT standards used.

### 3.2 Induction with theophylline

**Figure 2.**
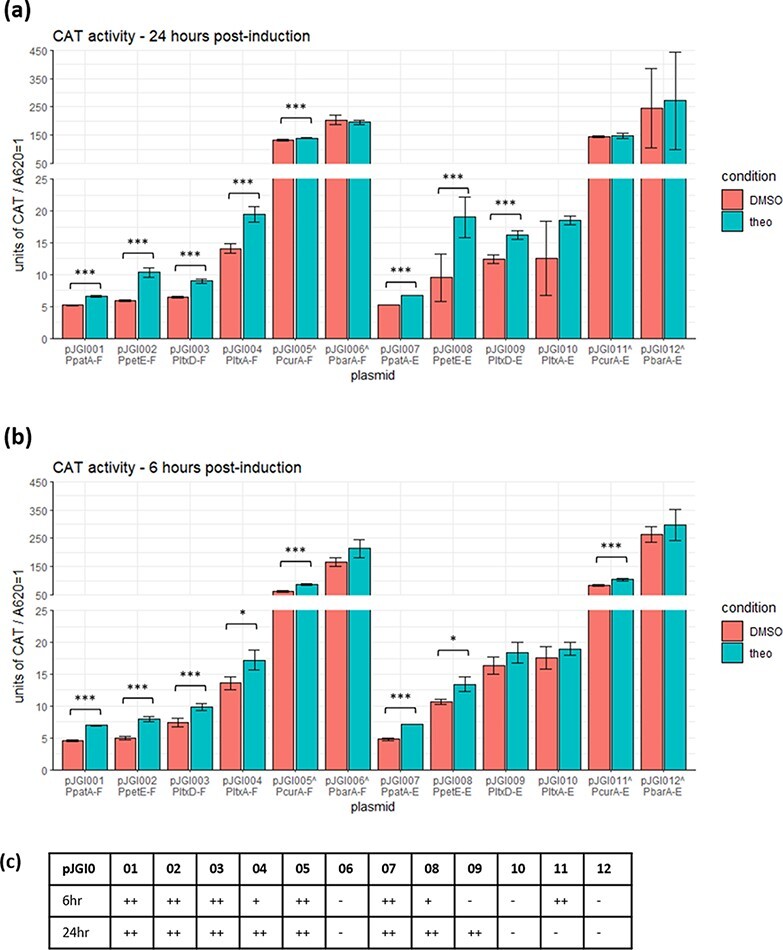
(a) Measured CAT activity from *Anabaena* 7120 cultures harvested 24 h after induction (3-ml sample). (b) Measured CAT activity from *Anabaena* 7120 cultures harvested 6 h after induction (1-mL sample). Production cultures grew for a week in BG-11(Nit) media before induction with 2 mM theophylline (dissolved in DMSO) or an equivalent volume of DMSO as a control. Colors correspond to induction conditions; error bars (in some cases, too close to the average to be clearly visible) show standard deviations from biological triplicates; ***P ≤ 0.001, *P ≤ 0.01. The ^^^ next to plasmid names indicates that for these, cell lysates were diluted 10× further for CAT activity to be in the readable range of the plate reader. (c) Summary of statistical significance between induced and uninduced CAT activities at both times.

Most promoter–riboswitch combinations were measured to contain significantly more CAT activity with theophylline induction than without (Figure 2a), and even the lowest producing constructs had over 5-fold more CAT activity than the background CAT activity measured from the negative controls (pPJAV361 and pPJAV561, Supplementary Figure S2). This suggests that the riboswitches did not completely suppress the production of CAT activity. With P*_patA_* (pJGI001 and pJGI007), P*_petE_* (pJGI002 and pJGI008) and P*_ltxD_* (pJGI003 and pJGI009), there was a significant increase in CAT activity from cultures that had been induced with theophylline with both riboswitches E (pJGI007-9) and F (pJGI001-3). With P*_ltxA_*, only riboswitch F resulted in a significant increase in measured CAT activity when comparing the induced and uninduced cultures. While pJGI005 (P*_curA_* with riboswitch F) did produce a statistically significant increase in measured CAT activity between the induced and uninduced cultures, this is attributable to the low variation between biological triplicates. As the difference in absolute values is small (1.05-fold), this construct would not be useful in experiments that required inducible protein production. The remaining constructs did not show a statistically significant increase in CAT activity upon induction with theophylline.

All of the strains containing a strong promoter (P*_curA_* and P*_barA_*) had exceedingly high measured CAT activity under both induced and uninduced conditions. The baseline production for all four high-producing promoter–riboswitch pairs is high enough that it is unlikely to have practical use as for induced protein production. However, they may find use as strong constitutive promoters to replace P*_glnA_*.

For P*_petE_*, P*_ltxD_* and P*_barA_* in particular, a greater amount of CAT activity was measured for both the induced and uninduced cultures with riboswitch E (pJGI008, pJGI009 and pJGI012) than riboswitch F (pJGI002, pJGI003 and pJGI006). This is similar to the results described by Ma *etal.* (23). Interestingly, P*_patA_* (pJGI001 and pJGI007), P*_ltxA_* (pJGI004 and pJGI010) and P*_curA_* (pJGI005 and pJGI011) were measured to contain similar amounts of CAT activity with both riboswitch E and riboswitch F.

### 3.3 Effect of riboswitch on promoter activity

Comparing the CAT activity measured when theophylline was included in the media consistently reduced the amount of measured CAT activity when compared to the promoters alone (Figure 2, Supplementary Figure S1). This suggests that the riboswitch attenuates translation, even in the presence of aptamer. A repeat of this experiment was conducted but with a 1-ml sample being examined for CAT activity at 6 h post-induction. At 6 h after induction with theophylline, the cultures showed a comparable amount of CAT activity to cells harvested 24 h after induction (Figure 2b). The 6-h timepoint data indicate that most of the CAT is produced in the first 6 h after induction, with a similar significance, if slightly lower absolute values compared to 24 h.

### 3.4 Removal of theophylline

**Figure 3.**
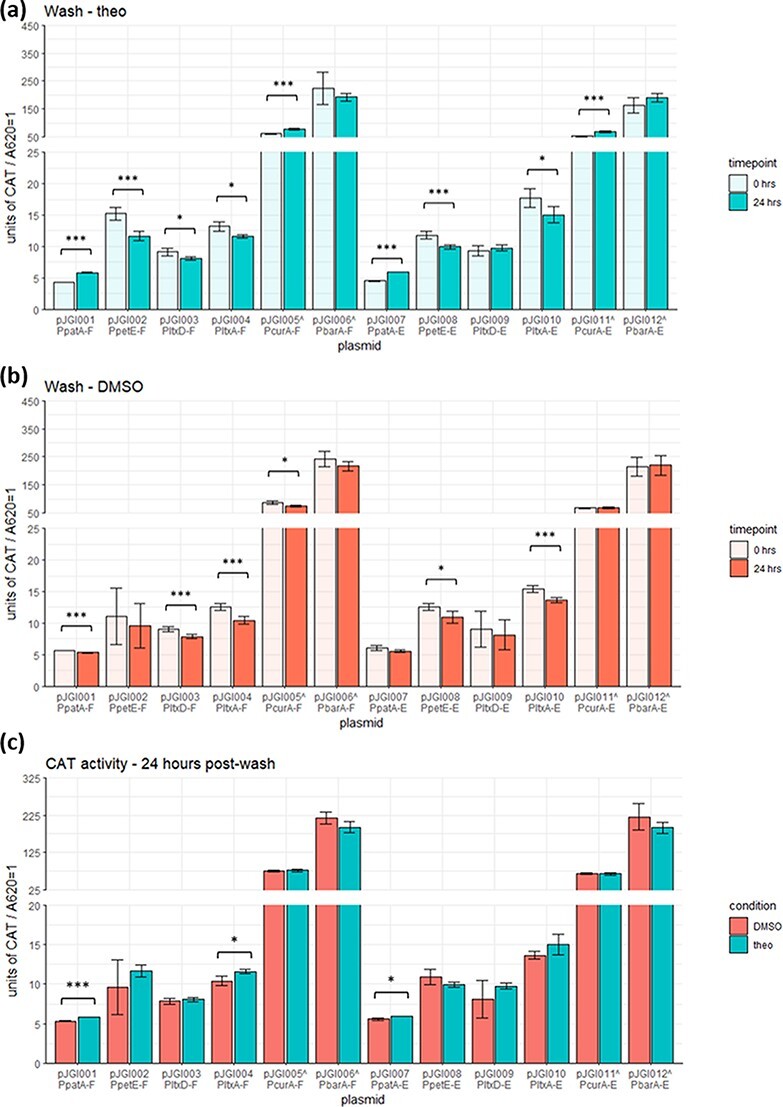
Measured CAT activity from *Anabaena* 7120 cultures harvested 24 h after washing with fresh media. Production cultures grew for a week in media with theophylline before cells were washed and allowed to grow for 24 h in fresh media, either with (a) or without (b) theophylline. (c) CAT activity between both conditions at 24 h compared. Colors correspond to fresh media conditions; error bars (in some cases, too close to the average to be clearly visible) show standard deviations from biological triplicates; *** *P* ≤ 0.001, *P ≤ 0.01. The ^^^ next to plasmid names indicates that for these, cell lysates were diluted 10× further for CAT activity to be in the readable range of the plate reader.

To examine whether these constructs could be used to create chemically conditional deletion mutants of essential genes in *Anabaena* 7120, we examined the CAT levels after growth in theophylline-containing media for 1 week followed by 24 h without theophylline. We expected a decreased level of CAT activity when washed with theophylline-free media, compared to a steady or increased level of CAT activity when washing with theophylline-containing media. We observed a small but fairly consistent reduction in measured CAT activity after washing for both theophylline and theophylline-free washing conditions (Figure 3aand b). The only constructs that showed a significant increase when theophylline was replaced were the ones containing P*_patA_* (pJGI001 and pJGI007) and P*_curA_* (pJGI005 and pJGI011) (Figure 3a). This differs from these same constructs when they were washed in theophylline-free media, where there was less or the same amount of CAT activity from 0 to 24 h after washing. Again, however, while the difference for high-producing P*_curA_* is statistically significant due to low variations between biological replicates, it is unlikely to be practically useful because of the high absolute values and relatively small differences between them. For all constructs, the CAT activity 24 h after washing was almost indistinguishable between conditions (Figure 3c). Much like with P*_curA_*, the statistical significance here is due to small biological variations, but the absolute values are very close together; pJGI001 (with a *P* < 0.001) shows an absolute difference of <0.5 CAT units per A_620_. This indicates that either CAT is not degraded after 24 h inside the cells or enough theophylline remains in the cells after washing and replacing the media to promote CAT synthesis. To provide evidence for these hypotheses, we examined the concentration of theophylline in the media after a 24-h incubation. Examination of four cultures (in biological triplicates) by the High-performance liquid chromatography analysis showed that the spent media generated by *Anabaena* 7120–containing pJGI001 contained 6.3 ± 0.5 nmoles in a 5-μl injection, while the media generated by cells containing pJGI007, pJGI006 and pJGI012 contained 5.8 ± 0.2, 4.7 ± 2.7 and 6.1 ± 0.6 nmoles of theophylline, respectively. Given that the initial concentration of theophylline was 2 mM, a total of 10 nmoles of theophylline would be expected as we detected no other peaks in the chromatogram, suggesting no decomposition of theophylline. This means that ∼40% of the added theophylline is either inside the cells or associated with the cell membrane, which supports the fact that despite washing three times with BG-11 media lacking theophylline, sufficient inducer remains in the cells to continue CAT production during the subsequent 24 h.

The results of this study show that a variety of cyanobacterial promoters can be combined with theophylline-responsive riboswitches for an inducible expression of heterologous proteins in *Anabaena* 7120. The weak and medium promoters tended to show a significantly higher production of CAT when induced with theophylline than when no theophylline was added. When paired with riboswitch F, these promoters showed a similar or lower baseline CAT production without theophylline induction than with riboswitch E. The constitutive strong promoters showed a much higher expression than the medium and weak promoters for both conditions and are not repressed by either riboswitch, with such a high baseline expression that it was even far above the induced expression of the medium and weak promoters. The washing experiment indicates that the weakest promoter, P*_patA_*, might have some sensitivity to the removal of theophylline, but none of the remaining promoters exhibited this trend. In future work, we also want to investigate the possibility of dual induction with both copper and theophylline, with the constructs containing P*_petE_*. Our preliminary experiment on this showed a high sensitivity to copper induction but little response to theophylline after a week of growth in copper-free media, unlike the response of pJGI002 (P*_petE_*–riboswitch F) and pJGI008 (P*_petE_*–riboswitch E) to copper in our usual copper-containing media (Supplementary Figure S4). The various performances of each of the promoter–riboswitch constructs under different conditions allow the researchers to select the one that best fits the purpose for their application. In addition, the lack of complete suppression of CAT production suggests that further optimization of the theophylline riboswitches would benefit the cyanobacterial research community and will be the subject of future research.

## Supplementary Material

ysab019_Supp

## Data Availability

Plasmids described in this manuscript are available from the corresponding author’s laboratory upon request.
