# Supplementary material for: Evaluation of inducible promoter–riboswitch constructs for heterologous protein expression in the cyanobacterial species Anabaena sp. PCC 7120
Source: Synth Biol (Oxf). 2021 Sep 1;6(1):ysab019. doi: 10.1093/synbio/ysab019 (PMC8546608; doi:10.1093/synbio/ysab019)
Supplement: ysab019_Supp [file ysab019_supp.zip › Supplementary Data-2021.07.10.pdf]

Supplementary Data for:

Evaluation of inducible promoter-riboswitch constructs for heterologous protein expression in the cyanobacterial species *Anabaena* sp. PCC 7120

Jessee Svoboda,[1] Brenda Cisneros,[2] Benjamin Philmus[1,2]*

[1] Department of Bioengineering, Oregon State University, Corvallis OR, 97331; [2] Department of Pharmaceutical Sciences, Oregon State University, Corvallis OR, 97331

*To whom correspondence should be addressed (benjamin.philmus@oregonstate.edu)

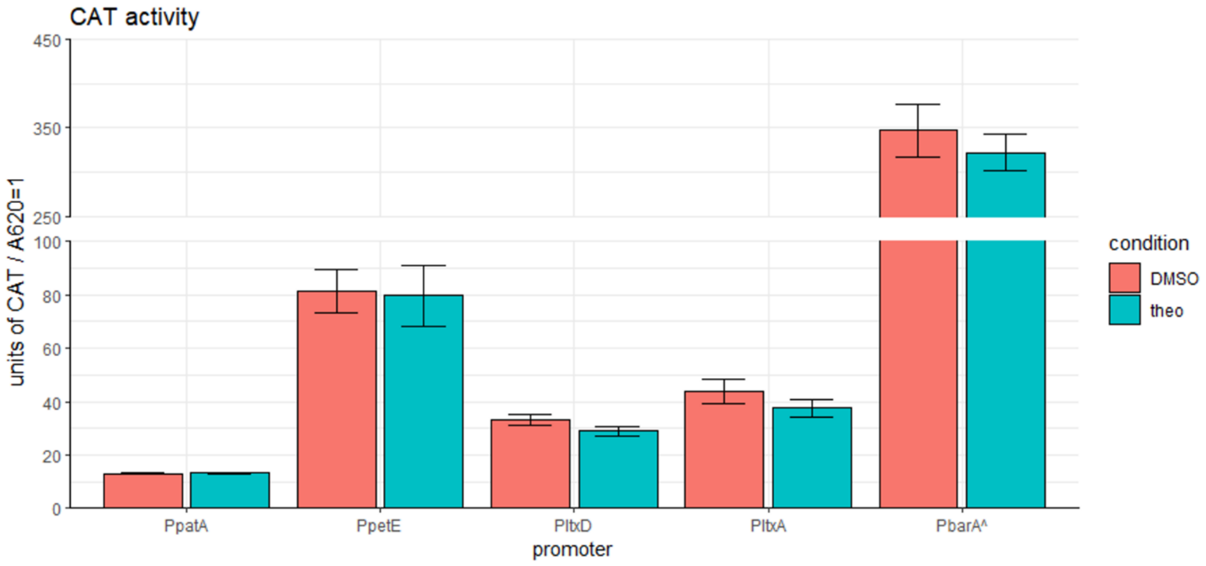

Supplementary Figure 1: Measured CAT activity from *Anabaena* 7120 cultures containing plasmids with CAT under the control of the promoters only (no riboswitches).(1) Cells were harvested 24 hours after induction. Production cultures grew for a week in BG-11(Nit) media before induction with 2 mM theophylline (dissolved in DMSO) or an equivalent volume of DMSO as a control. Colors correspond to induction conditions; error bars show standard deviations from biological triplicates. There was no statistical significance found between the DMSO and theophylline conditions for each promoter; a>0.01 for all. The ^ next to plasmid names indicates that for these, cell lysates were diluted 10x further for CAT activity to be in the readable range of the plate reader. All promoters show the same CAT activity, unaffected by the addition of theophylline, as expected. The strain with promoter P_{curA} did not grow, and therefore data is not included.

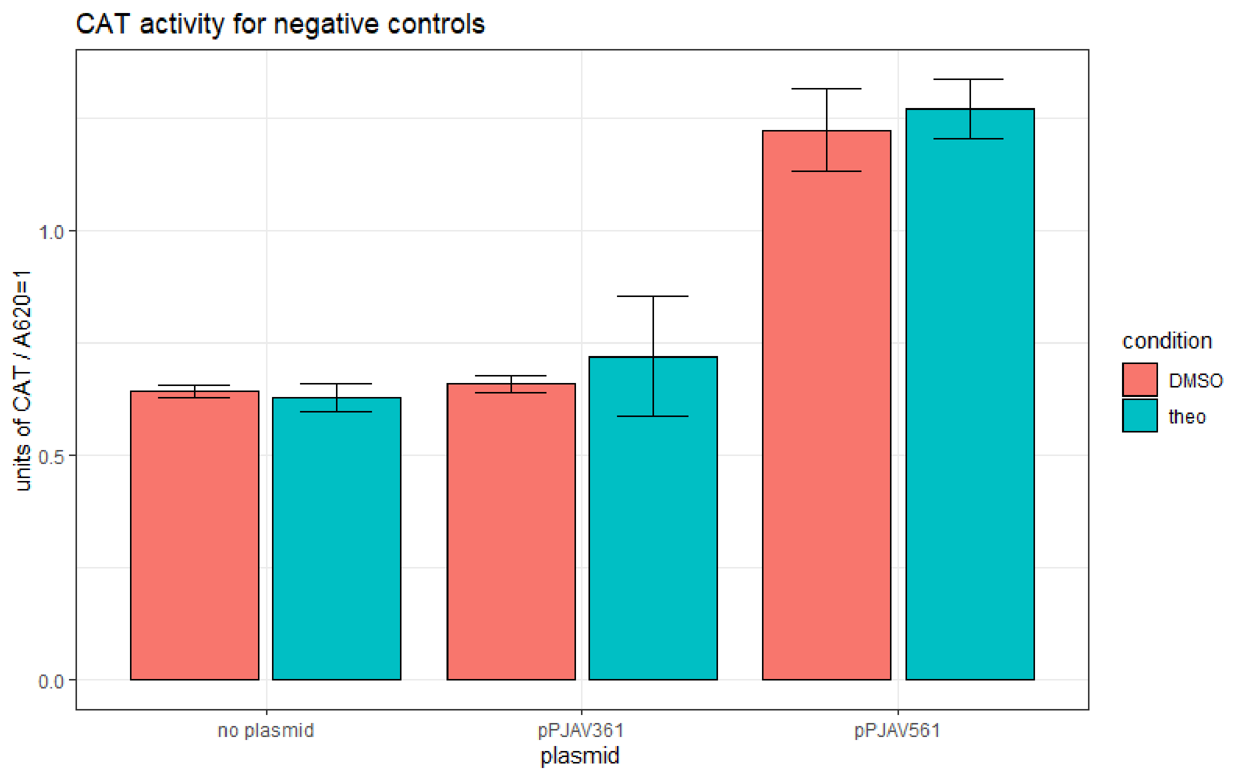

Supplementary Figure 2: Measured CAT activity from the negative control *Anabaena* 7120 cultures, containing no plasmids, the empty vector (pPJAV361), and the vector containing a promoter-less *cat* gene (pPJAV561), respectively.(1) Cells were harvested 24 hours after induction. Production cultures grew for a week in BG-11(Nit) media before induction with 2 mM theophylline (dissolved in DMSO) or an equivalent volume of DMSO as a control. Colors correspond to induction conditions; error bars show standard deviation from biological triplicates. There was no statistical significance found between the DMSO and theophylline conditions for each promoter; a>0.01 for all. The empty vector shows the same CAT activity as the plasmid-less strain, and the promoter-less vector had CAT activity just above that background level, unaffected by the addition of theophylline, as expected.

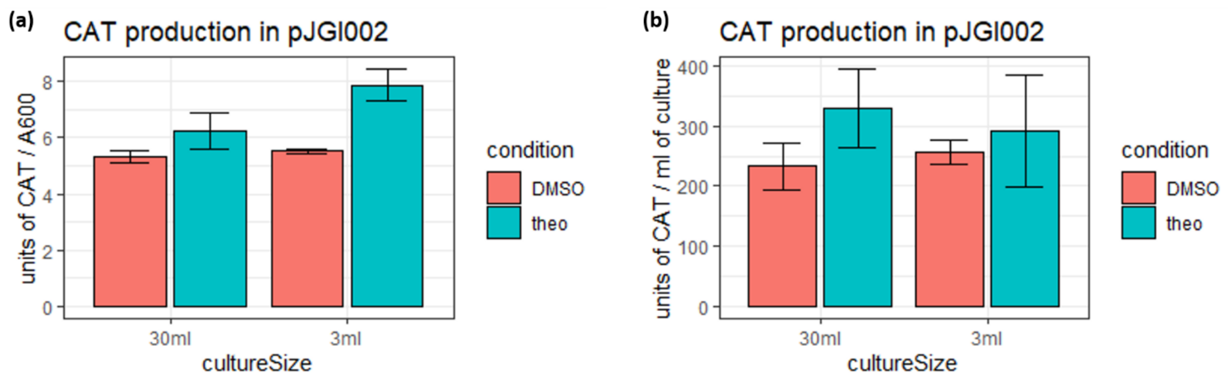

Supplementary Figure 3: Measured CAT activity from *Anabaena* 7120 cultures containing pJGI002, 3 mL samples. Cells were harvested 24 hours after induction. Production cultures grew for a week in either 3 mL or 30 mL of BG-11(Nit) media before induction with 2mM theophylline (dissolved in DMSO) or an equivalent volume of DMSO as a control. Cell lysates were normalized to A600=0.05 before the CAT assay. Both graphs visualize the same measurements from the same cultures. Colors correspond to induction conditions; error bars show standard deviations from biological triplicates.
(a) Results shown standardized per A_{600}=1.
(b) Results shown standardized per mL of culture.

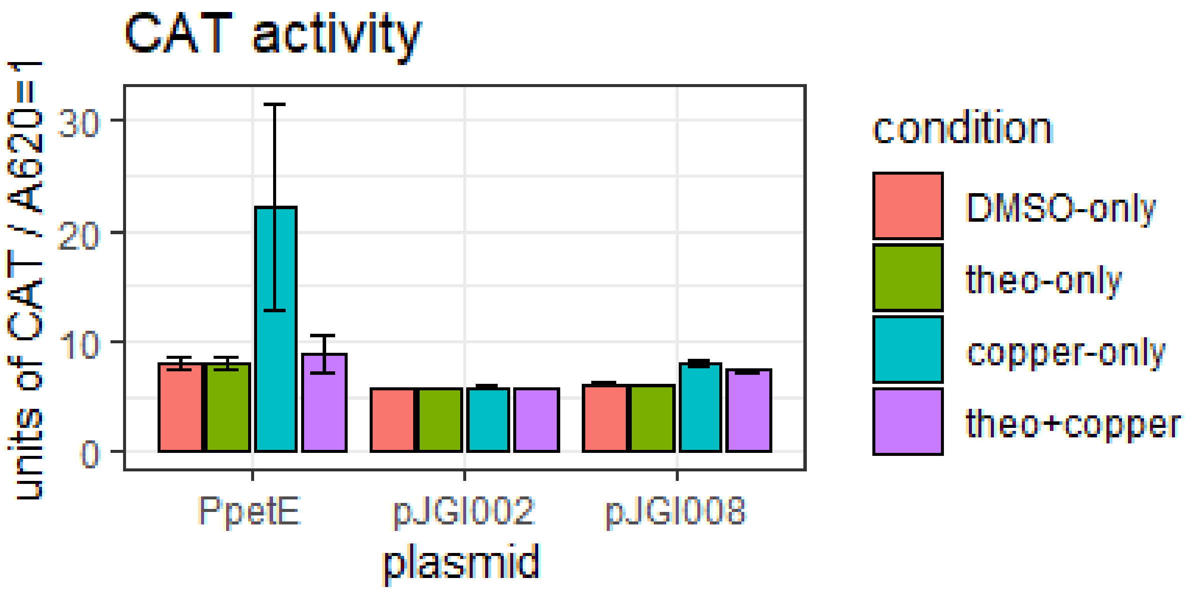

Supplementary Figure 4: Measured CAT activity from *Anabaena* 7120 cultures containing P_{petE}: the first one without a riboswitch, the second one with riboswitch F (pJGI002), the second one with riboswitch E (pJGI008). Cells were harvested 24 hours after induction. Production cultures grew for a week in 3 mL of BG-11(Nit) copper-free media before induction to 0.316uM copper (dissolved in DI water) and/or 2mM theophylline (dissolved in DMSO) or an equivalent volume of DMSO as a control. Cell lysates were normalized to A620=0.05 before the CAT assay. Colors correspond to induction conditions; error bars show standard deviations from biological triplicates.

References:

1. Videau P, Wells KN, Singh AJ, Gerwick WH, Philmus B. Assessment of Anabaena sp. Strain PCC 7120 as a Heterologous Expression Host for Cyanobacterial Natural Products: Production of Lyngbyatoxin A. *ACS Synth Biol.* **2016**, *5*(9):978–988.
